# Mechanisms of rhizosphere plant-microbe interactions: molecular insights into microbial colonization

**DOI:** 10.3389/fpls.2024.1491495

**Published:** 2024-11-06

**Authors:** Luna Yang, Xin Qian, Zeyu Zhao, Yaoyao Wang, Gang Ding, Xiaoke Xing

**Affiliations:** State Key Laboratory for Quality Ensurance and Sustainable Use of Dao-di Herbs, Institute of Medicinal Plant Development, Chinese Academy of Medical Sciences and Peking Union Medical College, Beijing, China

**Keywords:** rhizosphere, root colonization, molecular dialogue, root exudates, plant-microbe interactions

## Abstract

The rhizosphere, as the “frontline” of plant life, connects plant roots, rhizosphere microorganisms, and surrounding soil, plays a crucial role in plant growth and health, particularly in sustainable agriculture. Despite the well-established contribution of plant-microbe interactions to plant health, the specific molecular mechanisms remain insufficiently understood. This review aims to summarize the physiological adjustments and signal modulation that both plants and microorganisms undergo within this unique ecological niche to ensure successful colonization. By analyzing key processes such as chemotaxis, root attachment, immune evasion, and biofilm formation, we uncover how plants precisely modulate root exudates to either recruit or repel specific microorganisms, thereby shaping their colonization patterns. These findings provide new insights into the complexity of plant-microbe interactions and suggest potential directions for future research in sustainable agriculture.

## Introduction

The rhizosphere is the nutrient-rich zone of soil surrounding plant roots, which is characterized by intense microbial activity and diversity. It is regarded as one of the most intricate ecosystems on Earth, mediating subterranean interactions between plants and microorganisms (Goswami and Deka, 2022). These interactions, ranging from mutualism to parasitism, can be classified as forms of symbiosis (Plett and Martin, 2018; Du et al., 2021a). In mutualistic relationships, beneficial microorganisms such as rhizobia, mycorrhizae, endophytes (including plant growth-promoting microorganisms, PGPMs), and epiphytes establish positive interactions with plants, providing protection against stresses, enhancing growth, nutrient uptake, and improving soil conditions (Kiers et al., 2011; Zamioudis and Pieterse, 2012; Jin et al., 2019; Ding et al., 2022; Koprivova and Kopriva, 2022). In contrast, parasitism causes diseases that negatively affect plant growth (Venturi and Fuqua, 2013; Zhang et al., 2023).

**Figure 1:**
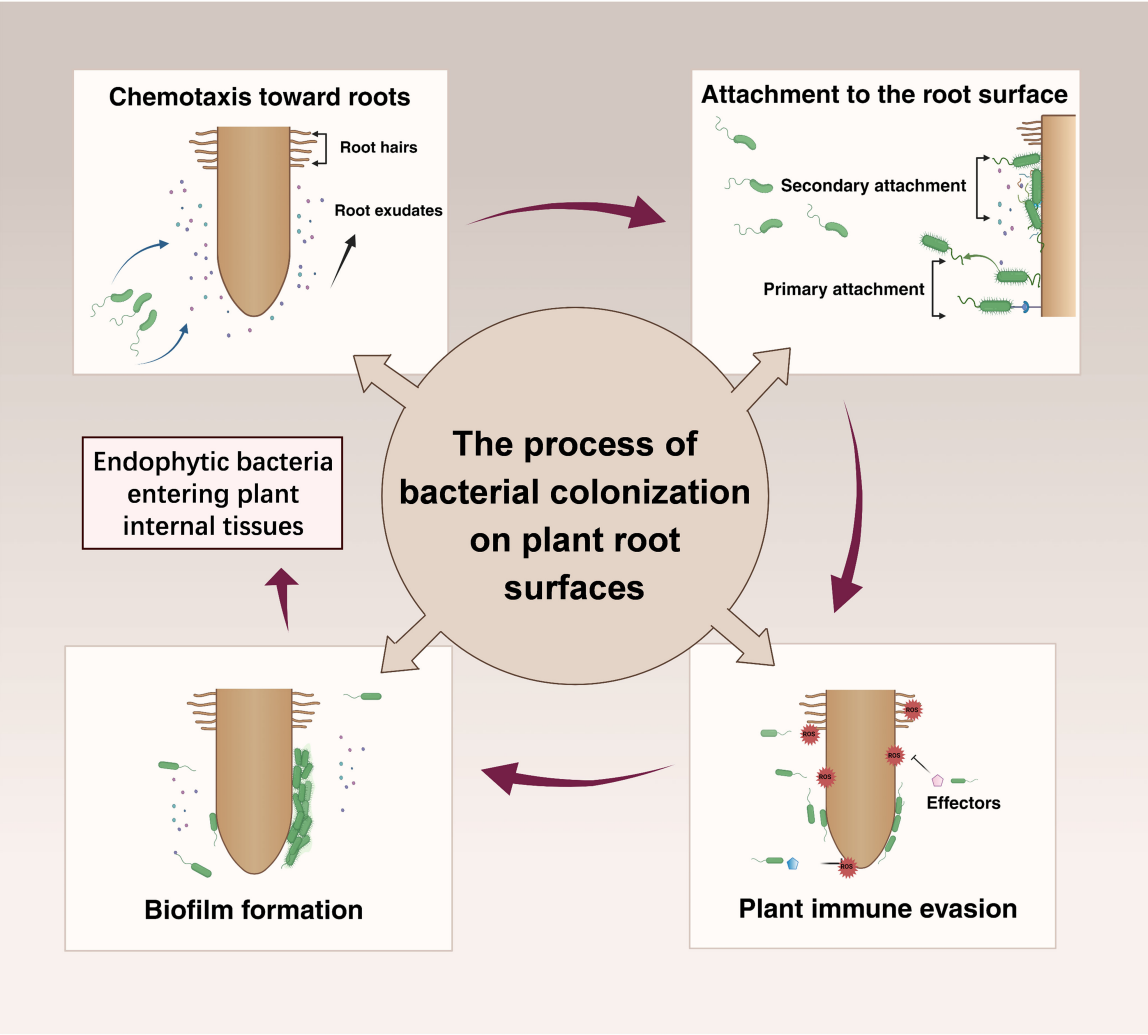
The colonization process of most bacteria can generally be divided into the following steps: chemotaxis, attachment, growth on the root surface, and biofilm formation. The chemotaxis and motility of microorganisms not only determine their movement toward the rhizosphere but also influence their initial positioning and migration of colonization sites. Following attachment to the root surface, microorganisms must overcome plant immune responses. Biofilm formation is essential for most bacteria colonizing the rhizosphere soil and root surfaces, and during this process, the bacteria utilize root exudates as a carbon source, which is a prerequisite for biofilm formation. Root exudates play a crucial role in influencing microbial colonization, and throughout the colonization process, a series of molecular dialogues occur between the interacting partners, indicating complex plant-microbe interactions.

“Rhizosphere colonization” refers to the process through which microorganisms establish themselves in the rhizosphere soil or on the root surface, thereby forming stable communities (Liu et al., 2024). Among these microorganisms, bacteria are particularly significant due to their widespread presence and profound influence on plant health and soil ecosystems (Liu et al., 2024). The root surface serves as a critical interface for direct interactions between bacteria and plants, making it essential to understand the mechanisms of bacterial colonization in this area to elucidate plant-microbe interactions. Consequently, this review focuses primarily on the process of bacterial colonization on root surfaces. The colonization process typically follows four key steps (**Figure 1**): (i) chemotactic signal recognition; (ii) attachment to the root surface; (iii) evasion of plant immune defenses; and (iv) biofilm formation on the root surface. Upon completing these steps, endophytic bacteria may subsequently enter plant internal tissues, facilitating more direct interactions with the plant (Dudeja et al., 2021; Mushtaq et al., 2023). Throughout the colonization process, both plants and bacteria adjust their physiological states, guided by molecular signals, which include chemical molecules and effector proteins. These signals, whether specific or non-specific, are perceived through receptors and signaling pathways, influencing bacterial colonization behavior (Badri et al., 2009). This “ molecular dialogue” is crucial for the successful colonization and the establishment of symbiotic relationships in the rhizosphere.

In this review, we comprehensively summarize the process of rhizosphere microbial colonization and its interactions with plants. By conducting an in-depth analysis of regulatory signals, such as chemical molecules and effector proteins, we delve into the “dialogue” mechanisms existing between plants and microorganisms. This review aims to provide a reference for future research and to advance the application of microorganisms in modern agriculture.

## Chemotactic signals emitted by plants roots

The chemotactic movement of soil microorganisms constitutes the fundamental basis of plant-microbe interactions, where root exudates frequently serve as communication molecules within this process (Chagas et al., 2018). Root exudates consist of significant amounts of carbon generated by plant photosynthesis, including low molecular weight compounds such as sugars, organic acids, and secondary metabolites (e.g., flavonoids, glucosinolates, and coumarins). These compounds make the rhizosphere a more active site for microbial colonization compared to bulk soil (Bais et al., 2006). Additionally, there are high molecular weight compounds like mucilage and proteins, despite their lower variability, they constitute the principal components of exudates (Chen and Liu, 2024). It is worth noting that due to the differential utilization and metabolic potential of various substrates among soil microorganisms, the chemical valence of rhizosphere exudates can drive positive or negative chemotactic movements in microbes (Feng et al., 2021; Knights et al., 2021).The following are detailed examples further illustrating the specific roles of root exudates in microbial chemotaxis:

1. Primary metabolites, such as sugars, amino acids, and organic acids, play essential roles in the rhizosphere by serving as carbon sources, chemoattractants, and chemical signals, thereby shaping microbial community structure and facilitating root colonization. Sugars are important carbon sources and colonization signals for rhizosphere microorganisms, influencing microbial community structure. In barley, key carbon compounds in root exudates help beneficial *Pseudomonas* adapt. A decrease in exudate diversity and higher glucose and fructose levels lead to more *Pseudomonas* (Pacheco-Moreno et al., 2024). Sugar molecules also positively influence the colonization of beneficial Gram-positive bacteria, particularly *Bacillus*. Additionally, sucrose, a widely present disaccharide, selectively shapes *Bacillus* in soil microbial communities. When *Bacillus subtilis* encounters sucrose exuded by Arabidopsis roots, it initiates the “levan detour” signaling cascade, activating solid surface motility (SSM), thereby enhancing its movement and colonization ability in the root environment (Tian et al., 2021).

In addition to sugars, amino acids in root exudates serve as key chemoattractants for various rhizosphere bacteria. For example, in *Sesbania rostrata*, the amino acids histidine, arginine, and aspartate act as chemoattractants for *Azorhizobium caulinodans*, not only facilitating bacterial chemotaxis but also upregulating the expression of genes involved in chemotaxis and motility. This dual role of amino acids underscores their importance in biofilm formation and root colonization (Liu et al., 2019). Moreover, in *Pseudomonas fluorescens* Pf0-1, the chemotaxis sensory proteins CtaA, CtaB, and CtaC have been identified as critical for amino acid-driven root colonization. Mutants lacking these proteins demonstrate reduced competitiveness, highlighting the pivotal role of amino acids as chemoattractants in the colonization process (Oku et al., 2012).

Organic acids further contribute to the rhizosphere’s ecological dynamics by regulating soil pH and increasing mineral solubility, thereby promoting nutrient uptake by plants. They also act as chemical signals during the chemotactic colonization of rhizosphere bacteria. Studies have shown that the high release of citric, pyruvic, succinic, and fumaric acids may account for the enrichment of *Comamonadaceae* (Wen et al., 2020). When *Arabidopsis thaliana* leaves are infected by the tomato pathogen *Pseudomonas syringae* pv. tomato DC3000, roots secrete L-malic acid (MA) as a signal to recruit beneficial rhizosphere bacteria *Bacillus subtilis* FB17 (Rudrappa et al., 2008).

2. Secondary metabolites can serve as attractants for specific microbial strains, promoting beneficial interactions within the rhizosphere. For instance, the secretion of coumarins by *Arabidopsis thaliana* under iron deficiency attracts beneficial microbes, which enhance plant growth by improving iron uptake (Harbort et al., 2020). On the other hand, secondary metabolites often act as negative chemotactic agents, playing a crucial role in defending plants against pathogens. For instance, glucosinolates (GSLs), secondary metabolites unique to cruciferous plants, are hydrolyzed to produce glucosinolate aglycones, which rearrange into isothiocyanates with potent antimicrobial activity (Wittstock and Burow, 2010; Siebers et al., 2018). Moreover, studies on flavonoids provide important insights into another mechanism by which secondary metabolites resist pathogens. In high-density microbial populations, intercellular communication triggers the production of virulence factors through a process known as quorum sensing (QS) (Fuqua et al., 2001). While quorum sensing is a mechanism of communication that facilitates cooperative behavior in many microbial communities, it may not function as a virulence strategy in all microbes. Research has demonstrated that catechins significantly reduce the expression of key QS regulatory genes lasI, lasR, rhlI, and rhlR in *Pseudomonas aeruginosa*, leading to decreased production of QS factors and forming a line of defense against pathogen attacks (Vandeputte et al., 2010). This suggests that secondary metabolites can not only directly kill pathogens but also inhibit pathogen virulence by disrupting intercellular bacterial communication, thereby exerting negative chemotactic effects on pathogenic bacteria.

The regulatory role of secondary metabolites in the rhizosphere extends beyond direct interactions with pathogens. For instance, the decomposition product of benzoxazinoids, 6-methoxy-benzoxazolin-2-one (MBOA), indirectly determines the composition of the next generation of rhizosphere microorganisms by altering the associated microbial community around the roots, thereby extending its regulatory effects into the soil (Hu et al., 2018). Benzoxazinoids can also influence soil microbial communities by regulating the release of secondary metabolites from rhizosphere plants, particularly flavonoids (Cotton et al., 2019). These findings further demonstrate that secondary metabolites, as signaling molecules, play a crucial role not only in facilitating communication between plants and microorganisms, but also in influencing interactions among microorganisms themselves. Additionally, these compounds significantly impact the soil environment within the rhizosphere. The regulatory effects of secondary metabolites are highly complex, depending on both the diversity of species that respond to these signals and the specific environmental conditions under which they function.

3. Volatile organic compounds (VOCs) primarily consist of terpenes, aromatic compounds, nitrogen-containing compounds, and fatty acid derivatives, as well as volatile plant hormones like methyl jasmonate and methyl salicylate as low molecular weight substances released by plant roots (Holopainen, 2004). VOCs, such as essential oils, have long been recognized for their broad antimicrobial activity, potentially playing a crucial role in mediating negative chemotaxis of pathogens. However, recent evidence indicates that VOCs can also serve as energy sources or signaling molecules, promoting the enrichment of beneficial bacteria by regulating bacterial growth, chemotaxis, and competitive ability, thereby exerting positive chemotactic effects (de la Porte et al., 2020; Sharifi et al., 2022). Due to their physicochemical properties, VOCs are more likely to diffuse throughout the soil layer, attracting bacteria at distances ranging from a few millimeters to as far as 12 centimeters from the roots, thereby regulating the structure of a broad microbial community (Schulz-Bohm et al., 2018). Schulz-Bohm et al. (2018) utilized an olfactometer system to verify that VOCs released by the roots of *Carex arenaria* could induce long-distance migration of soil bacteria toward the roots, explaining the crucial role of plant VOCs in facilitating long-distance plant-microbe interactions. Therefore, VOCs play a key role in long-distance interactions in the soil rhizosphere.

4. Complex polymers such as polysaccharides, proteins, and enzymes, known as high molecular weight (HMW) compounds, are also present in root exudates, although they are not easily utilized by soil microorganisms (Goswami and Deka, 2022). However, mucilage (polysaccharides) can form protective barriers, promote soil particle adhesion, and assist in the colonization of rhizosphere microorganisms (Read and Gregory, 1997). Additionally, proteins in root exudates, particularly those with enzymatic activity, enhance plant defense mechanisms through their constitutive release. For example, peroxidases can break down pathogen cell walls, induce defense signaling, and trigger plant immune responses, effectively curbing pathogen invasion. These enzymatic proteins can also recognize and recruit beneficial microorganisms, promoting the formation of symbiotic relationships and further enhancing plant health and stress resistance (Wen et al., 2007; De-la-Peña et al., 2010).

## Chemotactic signal reception and response by microorganisms

**Figure 2:**
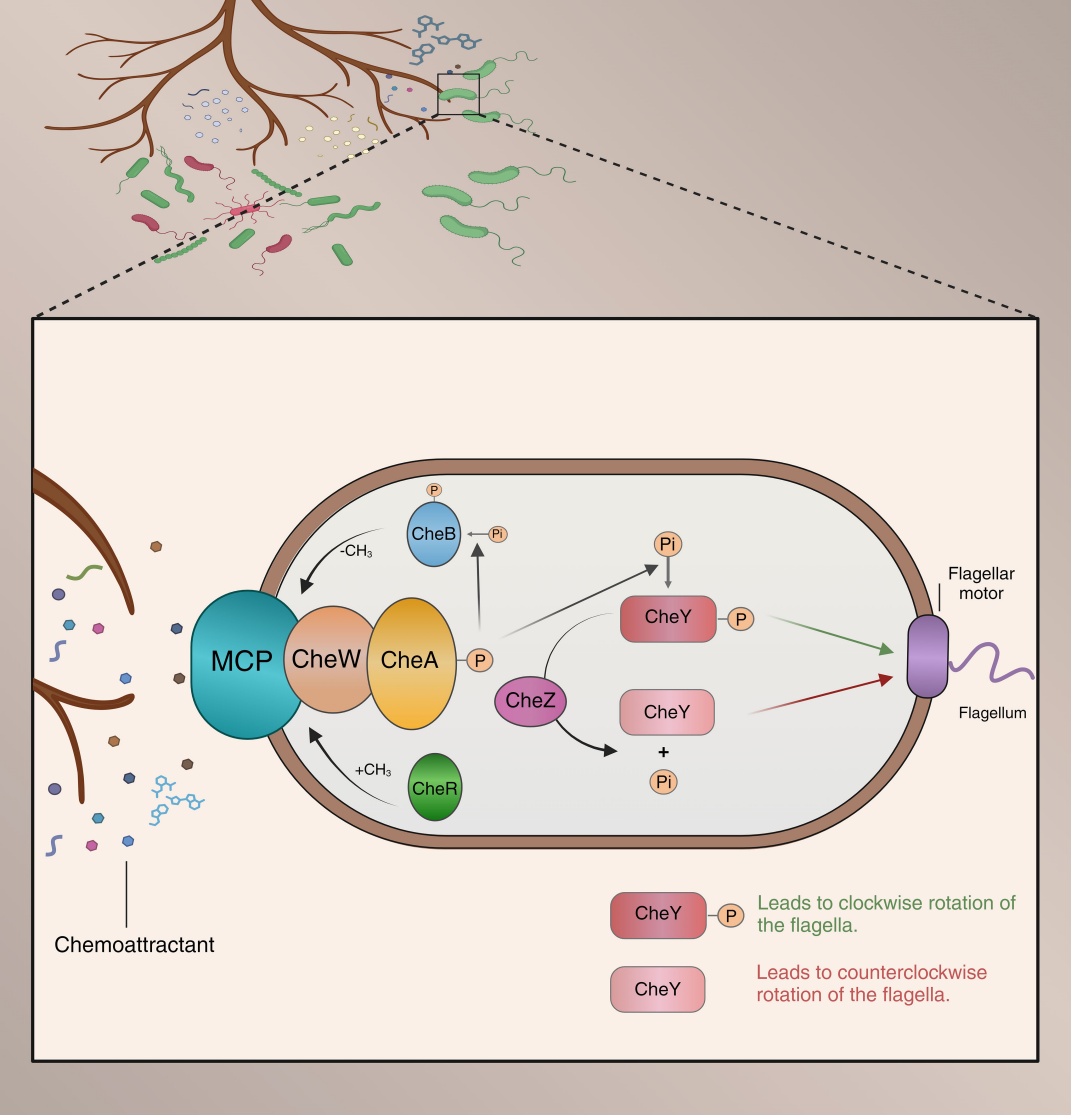
Model of microbial chemotaxis in the rhizosphere Chemical compounds secreted by plant roots attract beneficial bacteria while repelling harmful ones. These compounds are detected by transmembrane chemoreceptors on the bacteria, which regulate the autophosphorylation of CheA through the adaptor protein CheW. Once phosphorylated, CheA (CheA-P) phosphorylates the response regulator CheY. The phosphorylated CheY (CheY-P) binds to the flagellar motor, causing a switch in the direction of flagellar rotation from counterclockwise to clockwise, thereby altering the bacterial movement direction. The phosphatase CheZ dephosphorylates CheY-P, rapidly terminating the signal. CheB-P regulates methylation levels, working in conjunction with CheR to mediate the adaptive regulation of the chemoreceptors.

Upon perceiving chemotactic signals from plants, soil microorganisms move along a chemical gradient towards (attraction) or away from (repulsion) the signal source, engaging in chemotactic movement (Kearns, 2010). The chemotaxis system in bacteria is one of the most complex signal transduction systems in prokaryotes, with signaling and regulatory mechanisms relatively conserved across all bacteria. Specifically, bacterial chemoreceptor sensor proteins (MCPs) serve as receptors for chemotactic agents, sensing rhizosphere chemical effectors (Allard-Massicotte et al., 2016; Compton et al., 2018). Typically, MCPs are transmembrane proteins that form a ternary complex with histidine kinase CheA and coupling protein CheW (Sampedro et al., 2015). MCPs are located in the ligand-binding domain (LBD) of the cell membrane, which exhibits high structural variability to sense a wide range of chemical signals. For instance, the PGPR strain *Bacillus velezensis* SQR9 can sense various substances, including organic acids, sugars, amino acids, and sugar alcohols, primarily due to the pivotal role of its eight MCPs in mediating host interactions (Corral-Lugo et al., 2016; Feng et al., 2019; Tohidifar et al., 2020). When MCPs selectively recognize and bind specific root exudates, they trigger subsequent signal transduction and execution modules (Sampedro et al., 2015). Specifically, signal transduction regulates the autophosphorylation rate of histidine kinase CheA in a CheW-dependent manner, and phosphorylated CheA further influences the transphosphorylation of the CheY response regulator (McEvoy et al., 1999; Lacal et al., 2010). Ultimately, phosphorylated CheY interacts with motility proteins, mediating bacterial movement (Sampedro et al., 2015). The motility of rhizosphere bacteria manifests in various phenotypic forms, including swarming motility, swimming motility, gliding motility, and twitching motility (Kearns, 2010). These motility forms are driven primarily by flagellar rotation of individual bacterial cells, coordinated flagellar complex movement within the population, an extension of polar type IV pili, or passive surface spreading (Mattick, 2002; Mignot, 2007; Kearns, 2010). This sophisticated chemotaxis system enables bacteria to keenly sense the concentration gradient of extracellular signaling molecules, allowing them to adapt to changes in their surrounding environment. However, current research primarily focuses on bacterial responses to single chemotactic signals. In reality, both root exudates and bacterial MCPs exhibit high diversity, indicating that responses to composite signals may differ significantly from responses to single signals (**Figure 2**). Therefore, future research should focus more on bacterial responses to composite signals to gain a more comprehensive understanding of their behavior in complex environments.

## Bacterial attachment to root surfaces

Microorganisms move along a positive chemotactic signal gradient towards the host roots, selecting regions of the root with high concentrations of exudates as their colonization sites. Interestingly, microbial chemotaxis and motility play crucial roles in the selection of colonization sites on the roots. For instance, organic acids are strong attractants for *Azospirillum brasilense*, and *A. brasilense* mutants lacking chemotaxis fail to preferentially colonize the surface of the root maturation and elongation zones, which produce organic acids (O’Neal and Alexandre, 2020). The study also found that *A. brasilense* avoids the root tip region, which may produce toxic reactive oxygen species (ROS). This is because the root tip, being an area of active growth, generates hydrogen peroxide, which has a repellent effect on bacteria. Similarly, microorganisms must continuously migrate to avoid the immunologically active sites that change as the root develops (Tsai et al., 2023). These findings suggest that the host plays a significant regulatory role in bacterial selection of attachment sites on the roots.

Subsequently, microorganisms cease movement and adhere to the root surface, a critical step in achieving root colonization. Most agricultural microorganisms follow a common biphasic model for root attachment, which includes an initial attachment phase and a secondary attachment phase. During the initial attachment phase, rhizosphere bacterial cells form weak and reversible bonds with the root surface; in the secondary attachment phase, the bacteria’s attachment to the root surface becomes more secure, irreversible, and specific (Wheatley and Poole, 2018). Throughout the attachment process, microorganisms alter their physiological structures and secrete adhesive substances to successfully adhere. For example, bacteria may swing their flagella or pili to overcome electrostatic repulsion and secrete porins, outer membrane proteins, and lipopolysaccharides (LPS) as root surface adhesins (Berne et al., 2015; Knights et al., 2021). Additionally, bacteria secrete the signaling molecule di-AMP as an extracellular signal to regulate root attachment and biofilm formation, with *Bacillus subtilis* being a typical example (Townsley et al., 2018). This attachment is not merely a passive process; it involves active communication between the microbe and the plant. Plant surface molecules, such as lectins and arabinogalactan proteins, play a role in recognizing and binding microbial cells. This interaction is often species-specific, ensuring that only compatible microbes establish a symbiotic relationship (Imberty and Varrot, 2008). During this process, both the host and microorganisms change their lifestyles, achieving a balanced state through mutual regulation via molecular and physiological mechanisms.

After Pathogen-Associated Molecular Pattern (PAMP) recognition, PRRs located on the cell surface recruit the co-receptor BRI1-Associated Kinase 1 (BAK1), forming a receptor complex that activates downstream Receptor-Like Cytoplasmic Kinase (RLCK)-VII family members, such as Botrytis-Induced Kinase 1 (BIK1). Subsequently, RLCKs phosphorylate downstream targets, including Respiratory Burst Oxidase Homolog D (RBOHD) and Mitogen-Activated Protein Kinase Kinase Kinases (MAPKKKs), triggering a series of defense responses, such as ROS burst, calcium influx, Mitogen-Activated Protein Kinase (MAPK) activation, transcriptional reprogramming, and the production of plant hormones.

The figure also highlights the role of Type III secreted bacterial effectors (T3Es) in modulating plant defense mechanisms. Since some effectors interfere with multiple plant targets, they are represented multiple times in the figure.

## Bacterial immune recognition evasion

**Figure 3:**
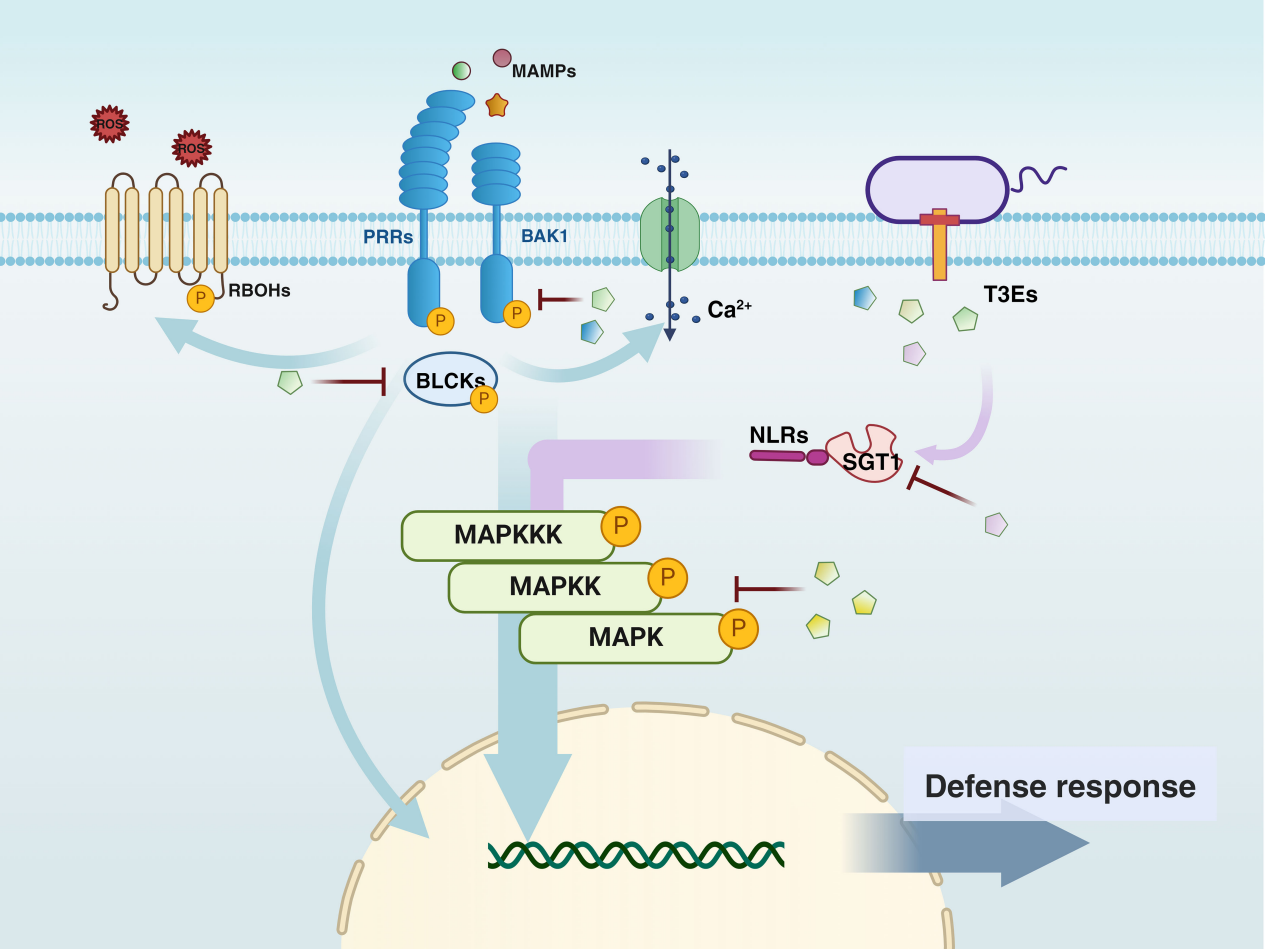
The figure illustrates the key components of PAMP-Triggered Immunity (PTI) and Effector-Triggered Immunity (ETI) signaling pathways in plant defense against bacterial pathogens, as well as the interconnections between these pathways. Arrows indicate the flow of defense signals, with light blue representing Pattern Recognition Receptor (PRR)-dependent signals and purple representing Nucleotide-binding Leucine-rich Repeat (NLR)-dependent signals. The convergence of these two signaling pathways is depicted by arrows that blend purple and light blue. PRR and NLR signals jointly regulate the plant immune response and interact at multiple critical points.

Once microorganisms attach to the root surface, overcoming the plant immune system becomes one of the crucial challenges for successful rhizosphere colonization. At this stage, microorganisms deploy a series of molecular strategies to adapt to and modify the plant environment, thereby alleviating the immune attacks they face during colonization (Jones and Dangl, 2006; Khavkin, 2021; Kong and Yang, 2023). The following chapter summarizes three strategies evolved by colonizing microorganisms (particularly pathogens and symbionts) in response to the evolutionary selection pressures exerted by plant pattern recognition receptors (PRRs) that activate host immunity (**Figure 3**).

Microorganisms possess widely conserved molecular patterns (MAMPs), such as flg22, elongation factor Tu (EF-Tu), cold shock proteins (CSP), lipopolysaccharides (LPS), chitin, phospholipids, and Nep1-like proteins, which can be recognized by different pattern recognition receptors (PRRs) in plants. The activation of immune signaling events, known as MAMP-triggered immunity (MTI), plays a crucial role in eliminating potential pathogenic infections and forms the first barrier to microbial colonization (Zipfel and Oldroyd, 2017). Microbial pathogens have evolved complex strategies to evade plant immunity, allowing them to effectively colonize roots. A key strategy involves avoiding detection by PRRs, which primarily includes altering the structure of MAMPs, degrading MAMP precursors, inhibiting their biosynthesis, or preventing MAMP release. For example, *Pseudomonas syringae* secretes the protease ArpA to degrade flagellin monomers, preventing the release of the immunogenic epitope flg22 and thus evading immune detection during root colonization (Pel et al., 2014).

Beneficial microorganisms also employ similar strategies to evade recognition by the plant immune system. However, unlike pathogenic microorganisms, which rapidly adjust the properties of MAMPs, the immune evasion of beneficial microorganisms is more often achieved through the positive selection of PRRs over long-term evolution. For instance, variations in the flagellin protein sequence of the nitrogen-fixing symbiont *Sinorhizobium meliloti* result in its inability to activate immune responses in Arabidopsis (Felix et al., 1999). Similarly, the FLS2 receptor in grapevines has a weaker recognition of the flg22 epitope derived from beneficial *Bacillus subtilis* compared to that from pathogenic *Pseudomonas syringa*e and *Xanthomonas campestris*, leading to a significantly reduced immune response (Trdá et al., 2014). Beneficial symbiotic microorganisms can also avoid detection by creating environments unfavorable to immune recognition. For example, *Pseudomonas capeferrum* WCS358 lowers environmental pH by secreting organic acids, thereby inhibiting plant recognition of flg22 (Yu et al., 2019). However, current research on immune evasion primarily focuses on flg22, and future studies should extend to other MAMPs to uncover the mechanisms by which rhizosphere microorganisms evade immune recognition during colonization. In addition to MAMPs, mutualistic symbiotic microorganisms also secrete symbiosis-related molecules, which are recognized by host symbiotic receptors and initiate signal transduction (Zipfel and Oldroyd, 2017). For example, Nod factors from rhizobia are lipochitooligosaccharides similar in structure to MAMPs like chitin and peptidoglycan, and they initiate the symbiotic process of rhizobia (Oldroyd, 2013). Interestingly, many symbiotic molecules from beneficial microorganisms seem to suppress the local immune responses triggered by MAMPs in roots (Gourion et al., 2015). For instance, Nod factors from rhizobia significantly suppress MAMP-induced immune responses in both leguminous and non-leguminous plants like Arabidopsis, leading to a significant reduction in the levels of homologous PRR proteins on the cell membrane (Liang et al., 2013; Gourion et al., 2015).

Although the mechanisms by which plant immunity regulates pathogenic and beneficial microorganisms have been elucidated (Yang et al., 2022), microorganisms in the rhizosphere often exist in community forms, which means plants need to sense the presence of entire communities. The FLS2 receptor in plants plays a crucial role in this process, acting as a community sensor to detect the relative proportions of danger signals (canonical peptides) from pathogens and signals (evading and modulating peptides) from safe microorganisms. This sensing mechanism influences the assembly of specific pathogenic and symbiotic microbial communities, enabling the plant to distinguish between friends and foes (Colaianni et al., 2021). This discovery challenges the traditional view that the flg22-FLS2 complex only drives immune signal transduction, showcasing the complexity and subtlety of plant regulation in immunity and symbiosis.

## Bacterial effectors interfere with immune signaling

Bacteria can secrete effectors, primarily composed of effector proteins, into the apoplast or deliver them into plant cells through specialized secretion systems (Jones and Dangl, 2006). These effectors bypass plant MTI by targeting immune signal transduction components. Research into the mechanisms of pathogen effectors provides important insights into how bacterial effectors suppress plant immune responses by interfering with key immune signaling molecules. Specifically, BRI1-Associated Kinase 1 (BAK1) and Bacterial-Like Cytoplasmic Kinases (BLCKs) are major targets for various pathogen effectors (Wang et al., 2022). BAK1 acts as a co-receptor for multiple pattern recognition receptors (PRRs) and plays a critical role in immune signal transduction induced by various MAMPs (Yasuda et al., 2017). For example, the pathogen *Phytophthora sojae* effector PsAvh110 specifically binds to the soybean heterochromatin protein GmLHP1-2, disrupting its assembly with the transcriptional complex GmPHD6, thereby inhibiting the expression of a set of immunity-related genes, including the BRI1-associated receptor kinase GmBAK1-3 (Qiu et al., 2023). Botrytis-induced Kinase 1 (BIK1), a member of the BLCK-VII subfamily, is a core regulator of plant immunity, located downstream of PRR and BAK1 signaling pathways, and is responsible for transmitting signals to downstream MAPK cascades, initiating a series of cellular defense responses, and coordinating the plant’s defensive actions (Ma et al., 2020). The type III secretion effector RipAC from the bacterium *Ralstonia solanacearum* targets the plant E3 ubiquitin ligase PUB4, suppressing pattern-triggered immunity (PTI) and leading to the degradation of the critical immune kinase BIK1 (Yu et al., 2022). Additionally, within plant cells, Nucleotide-binding Leucine-rich Repeat-like receptors (NLRs) recognize effectors either directly or indirectly, activating effector-triggered immunity (ETI), which triggers a more robust immune response (Gourion et al., 2015). Pathogen effectors also disrupt multiple stages of ETI signal transduction. As mentioned earlier, the *Ralstonia solanacearum* effector RipAC not only drives BIK1 degradation but also interferes with the phosphorylation of SGT1 (a regulator of NLR accumulation or activation) and its interaction with MAPKs (Yu et al., 2020). The multifaceted mechanisms by which RipAC regulates plant immune responses suggest that the targets of effectors are often not singular, reflecting the complex regulatory methods and further emphasizing the multi-layered strategies pathogens employ to evade plant immunity. It is noteworthy that pathogens can evade NLR receptor recognition by regulating effector gene expression, such as altering promoter regions or applying epigenetic modifications. However, as this involves more epigenetic aspects, it will not be detailed in this article, but relevant content can be found in other literature (Wang et al., 2022).

Activation of the MAPK cascade is an early signal transduction event shared by both PTI and ETI and plays a key role in regulating immune outputs such as callose deposition, hormone production, and transcriptional reprogramming (Tzipilevich et al., 2021). This critical node is also often targeted by pathogens to weaken host immunity. For instance, the *P. syringae* T3Es effectors HopA1 and HopF2 inactivate MAPKs and their upstream kinases through different mechanisms. HopA1 acts as a phosphothreonine lyase, physically inactivating MPK3 and/or MPK6, while HopF2 inactivates MAPK kinase 5 (MKK5) through ADP-ribosylation (Zhang et al., 2007; Wang et al., 2010). Additionally, the *P. syringae* effector AvrB interferes with plant hormone signaling by activating MAPK MPK4, thereby negatively regulating pathogen defense (Cui et al., 2010). These findings demonstrate that the regulation by effector proteins is often not singular; they frequently activate different mechanisms to enhance their effects. The *Phytophthora infestans* RXLR effector PITG20303 further emphasizes this conclusion, targeting the stabilization of the potato MAPK cascade protein StMKK1, suppressing PTI, promoting pathogen colonization, and evading recognition by the resistance protein Rpi-blb2, thereby negatively regulating the plant’s PTI response (Du et al., 2021b).

Similar to plant pathogens, symbiotic rhizobia in legumes use a type III protein secretion system (T3SS) to deliver toxin-like type III effectors (T3Es) into plant cells, utilizing multiple effectors to suppress plant immune activation. These T3Es not only inhibit the immune response in legumes but also stimulate the production of nodulation signals, which are crucial for establishing symbiosis (Ferguson et al., 2010; Oldroyd, 2013). The NopL effector secreted by *Sinorhizobium* sp. strain NGR234 is a typical example; its serine-proline motif is essential for its effector activity during symbiosis, and over-phosphorylated NopL mutants show significantly reduced activity during symbiosis (Ge et al., 2016). The mechanism by which NopL suppresses immune responses by interfering with the plant MAPK cascade is gradually being uncovered. For example, NopL forms a complex with the tobacco MAP kinase SIPK and is phosphorylated by SIPK *in vitro*, thereby suppressing MAP kinase signal-mediated defense responses. Additionally, NopL mimics MAPK substrates and interferes with their signal transduction, inhibiting host cell death and premature nodule senescence (Zhang et al., 2011). Thus, it can be inferred that NopL suppresses plant defense responses by interfering with the MAPK signaling process or its downstream events. Recent studies have shown that, unlike the strategy of pathogen and rhizobial symbionts that rely on T3SS to inject highly specific effector proteins, beneficial rhizosphere symbionts primarily interfere with MTI signal transduction through non-specific extracellular mechanisms. The synthetic communities (SynComs) constructed from diverse MTI-suppressing strains in Arabidopsis roots primarily inhibit MTI by modulating specific and conserved parts of the host immune system. Gene screening further revealed that although *Dyella japonica* MF79 carries T3SS genes, its potent MTI-suppressing function mainly relies on the type II secretion system (T2SS), while T3SS is not essential for its inhibitory activity (Ökmen et al., 2013).

Although the mechanisms of action for individual bacterial effectors have been extensively studied, the synergistic effects of multiple effectors within the same pathogen and the temporal and spatial regulation by different pathogens in secreting effectors at various infection stages to evade host defense systems remain unclear. These complex molecular dialogues and regulatory networks require further research to elucidate their synergistic mechanisms and interactions in plant immunity.

## Microbial tolerance to immune responses

Activated immune signaling cascades ultimately trigger a series of powerful immune responses, including the secretion of proteases and inhibitors, the release of antimicrobial molecules, and bursts of ROS. Plants with activated defense responses synthesize and secrete various defense molecules and proteases, generating resistance to pathogenic microorganisms through different mechanisms, thereby altering the rhizosphere environment (Jones and Dangl, 2006; Wang et al., 2022). Meanwhile, symbiotic microorganisms cleverly evade these immune responses and even modify the environment to successfully promote their survival and proliferation. To successfully infect host plants and overcome their defense mechanisms, rhizosphere microorganisms have developed various strategies, which can be categorized into active neutralization and passive adaptation.

Active Neutralization Strategies include directly intervening in and regulating host plant immune responses by inhibiting the production of plant defense substances and secreting specific inhibitors. For example, the effectors AVRblb2 from *Phytophthora infestans* and Avh240 from *Phytophthora sojae* target plant PLCP C14 and aspartic protease AP1, preventing the secretion and activation of these defense proteins (Bozkurt et al., 2011; Guo et al., 2019). Similarly, the Kazal-like protease inhibitor EPI1 secreted by *P. infestans* can inhibit the plant resistance protease P69B, thereby hindering the maturation of immunogenic peptides (Paulus et al., 2020; Wang et al., 2021). In addition, pathogenic microorganisms suppress ROS production or interfere with their transport in host plants as a key strategy to actively neutralize host defense mechanisms. Host plants primarily produce ROS through peroxidases and membrane-bound NADPH oxidases. To suppress ROS, pathogenic microorganisms secrete various effectors. For example, *Ustilago maydis* effector Pep1 directly inhibits maize peroxidase POX12 (Hemetsberger et al., 2012), while *Phytophthora parasitica* effector PpE18 weakens the immunity of *Nicotiana benthamiana* by inhibiting the ROS-scavenging function of NbAPX3-1 and interfering with its interaction with NbANKr2 (Cao et al., 2024). Similarly, the *Phytophthora sojae* effectors Avr3b and CRN78 reduce ROS accumulation and transport by disrupting NADH availability and phosphorylating aquaporin PIP2;2 (Ai et al., 2021).

Passive Adaptation Strategies involve setting up biological barriers and altering physiological structures to adapt to plant defenses. Pathogenic microorganisms evade host immune responses through post-translational modifications of effector molecules. For instance, the virulence factor XEG1 produced by *Phytophthora sojae* undergoes glycosylation modifications to evade degradation by the host protease AP5 (Xia et al., 2020). Furthermore, pathogenic microorganisms can detoxify antimicrobial compounds synthesized by plants, such as the conversion of toxic α-tomatine into less toxic derivatives by the enzyme secreted by *Cladosporium fulvum* (Ökmen et al., 2013). Microbial pathogens have also developed various strategies to evade oxidative stress during infection. A typical example is that bacterial pathogens can produce extracellular polysaccharides to form protective layers, shielding themselves from oxidative stress induced by the host (Fones and Preston, 2012).

Similar to pathogenic microorganisms, beneficial microorganisms employ both active and passive adaptation strategies to cope with ROS bursts in plants, allowing them to successfully colonize the rhizosphere. Enhancing tolerance is a key strategy for responding to activated root immune responses, such as ROS bursts. For example, the colonization of beneficial bacterium *Bacillus velezensis* triggers plant immune responses and ROS production, which in turn stimulate bacterial auxin production, reducing ROS toxicity and playing a crucial role in root colonization (Tzipilevich et al., 2021). *Bacillus subtilis* SQR9 induces oxidative bursts in cucumber and Arabidopsis through its flg22 homologs, demonstrating high H_2_O_2_ tolerance and the ability to suppress oxidative bursts, with the ResD-ResE signal transduction system in SQR9 playing a key role in tolerating plant oxidative stress and root colonization (Zhang et al., 2021). Additionally, recently revealed spatial adaptation mechanisms indicate that plant cortical, endodermal, and root hair cells exhibit different defense capabilities, and beneficial microorganism *Pseudomonas simiae* WCS417 selects colonization sites with lower stress, demonstrating a spatially host-immune-driven adaptation strategy (Verbon et al., 2023).

Active Regulation: For example, the NopM effector from *rhizobium* NGR234 significantly suppresses flg22-induced ROS bursts when expressed in *Nicotiana benthamiana*, blocking ROS-related defense responses (Xin et al., 2012). This demonstrates that beneficial microorganisms can suppress transient ROS bursts by secreting effectors, cleverly interfering with plant immune responses, and showcasing their strategies and potential in actively regulating plant immunity.

Through the detailed analysis of the strategies employed by beneficial and pathogenic microorganisms in coping with plant immune systems, we can see that both types of microorganisms use many similar strategies in immune recognition evasion, immune signal interference, and immune response suppression. However, their ultimate goals are entirely different: pathogenic microorganisms aim to overcome plant immune systems to cause infection, while beneficial microorganisms regulate plant immune systems to establish symbiotic relationships.

## Biofilm formation and stable colonization

Biofilm formation is a key strategy employed by microbes to ensure stable colonization on the root surface. Biofilms are complex communities of microorganisms embedded in a self-produced extracellular matrix, which provides protection against environmental stresses and host immune responses (Flemming et al., 2023). This matrix mainly consists of polysaccharides, proteins, amyloids, lipids, extracellular DNA (eDNA), as well as membrane vesicles and humic-like microbially derived refractory substances (Flemming et al., 2023). The properties of these polymers confer resistance or antimicrobial tolerance to biofilms under certain adverse conditions. Beneficial rhizosphere bacteria first halt their motility and then form biofilms, a process generally controlled by one or more of their own transcriptional regulators (Arnaouteli et al., 2021; Nie et al., 2022; Ivanova et al., 2023). However, increasing evidence suggests that this process is also co-regulated by global transcriptional regulators mediated by environmental factors such as root exudates. For example, *Bacillus subtilis* colonizes Arabidopsis roots by forming biofilms dependent on extracellular matrix genes, a process triggered by plant polysaccharides and transmitted through the regulation of the phosphorylation state of the master regulator Spo0A (Beauregard et al., 2013). Interestingly, these polysaccharides not only serve as signals but also as sugar sources for synthesizing the extracellular polysaccharides (ESP) in the matrix, demonstrating the critical role of external regulation in bacterial root colonization (Beauregard et al., 2013). Similarly, phenolic compounds in root exudates attract and promote endophyte biofilm formation on the host root surface, facilitating colonization. After colonization, endophytes enhance the host’s intrinsic defense mechanisms by increasing the levels of phenolic antimicrobial substances in the rhizosphere exudates, significantly boosting the plant’s resistance to pathogen attacks (Ray et al., 2018). Root exudates also influence biofilm formation in pathogenic bacteria. *Pseudomonas aeruginosa* strains PAO1 and PA14 can infect the roots of Arabidopsis and sweet basil, forming biofilms and causing plant death. Inducing the secretion of rosmarinic acid (RA) from the host roots before infection or the exogenous addition of RA effectively counters *Pseudomonas aeruginosa* (Walker et al., 2004). Notably, although chemotaxis and biofilm formation are two distinct processes in rhizosphere colonization, the same molecule secreted by the host can regulate chemotaxis or biofilm formation in rhizosphere microorganisms depending on its concentration (Zhang et al., 2014; López-Farfán et al., 2019). This dose-dependent signaling is very common in the regulation of biofilm formation and chemotaxis in rhizosphere bacteria, highlighting the critical role of concentration gradients of environmental signals in microbial behavior.

## Summary and future perspectives

Previous research on microbial colonization processes has predominantly focused on isolating beneficial strains from rhizosphere bacteria. However, future studies should prioritize the regulation of these colonization processes to effectively mitigate pathogen invasion and enhance the efficiency of beneficial microbes in field applications. Potential regulatory strategies may involve manipulating the composition of root exudates, modulating microbial signaling pathways, or employing gene-editing technologies to optimize the colonization potential of beneficial microorganisms. By elucidating the interaction mechanisms between various microbial functional groups in the rhizosphere, we can enhance microbial symbiosis and competitive dynamics under conditions that closely mimic natural environments, thereby promoting plant health and advancing agricultural sustainability.

Future research should focus on investigating microbial colonization mechanisms within complex ecological contexts, examining the roles of soil physicochemical properties, microbial community structure dynamics, and interspecies interactions in shaping plant health. A multi-layered, systems-based approach that integrates the soil microenvironment and the ecological dynamics of microbial communities will provide a more precise theoretical foundation for agricultural management practices. This, in turn, will enhance crop resilience, optimize yield, and contribute robustly to sustainable agricultural development.
